# Supplementary material for: LC-HRMS Screening and Identification of Novel Peptide Markers of Ricin Based on Multiple Protease Digestion Strategies
Source: Toxins (Basel). 2019 Jul 5;11(7):393. doi: 10.3390/toxins11070393 (PMC6669667; doi:10.3390/toxins11070393)
Supplement: Supplementary file 1 [file toxins-11-00393-s001.pdf]

Supplementary Materials: LC-HRMS Screening and Identification of Novel Peptide Markers of Ricin Based on Multiple Protease Digestion Strategies

Long-Hui Liang, Chang-Cai Liu, Bo Chen, Long Yan, Hui-Lan Yu, Yang Yang, Ji-Na Wu, Xiao-Sen Li and Shi-Lei Liu

Table S1. The specific ricin tryptic peptides under digestion with denaturation and reduction.

T#&chain	Amino acid sequence	(M + H)+	(M + 2H)2+	(M + 3H)3+	(M + 4H)4+
T2A-glyc*	QYPIINFTTAGATVQSYTNFIR	3675.6946	1838.3509	**1225.9031**	919.6791
T7A	VGLPINQR	896.5312	**448.7692**	299.5152	-
T8A	FILVELSNHAELSVTLALDVTNA YVVGYR	3206.7095	1603.8584	1069.5747	**802.4328**
T9A	AGNSAYFFHPDNQEDAEAITHL FTDVQNR	3307.5038	1654.2555	1103.1728	**827.6314**
T10A	YTFAFGGNYDR	1310.5800	**655.7936**	437.5315	-
T11A	LEQLAGNLR	1013.5738	**507.2905**	338.5294	-
T12A	ENIELGNGPLEEAISALYYYSTGG TQLPTLAR	3440.7219	1720.8646	**1147.5788**	860.9359
T13A	SFIICIQMISEAAR	1581.8127	**791.4100**	527.9424	-
T23A	FSVYDVSILIPIIALMVYR	2212.2450	1106.6261	**738.0865**	553.8167
T3B	NGLCVDVR	875.4403	**438.2238**	292.4850	-
T5B	FHNGNAIQL WPCK	1584.7740	792.8906	**509.9223**	396.9494
T6B	SNTDANQLWTLK	1390.6961	**695.8517**	464.2369	-
T10B	CLTTYGYSPGVYVMIYDCNTAA TDATR	2949.3103	1475.1588	**983.7750**	737.5791
T11B-glyc*	WQIWDNGTIINPR	2991.2987	1496.1530	**997.7710**	748.5801
T12B	SSLVLAATSGNSGTTLTVQTNIY AVSQGWLPTNNTQPFVTTIVGL YGLCLQANSGQVWIEDCSSEK	6933.4237	3467.2155	2311.8127	1734.1119
T14B	DNCLTSDSNIR	1237.5477	**619.2775**	413.1874	-
T18B	NDGTILNLYSGLVLDVR	1862.0018	931.5045	**621.3388**	-
T20B	QIILYPLHGDPNQIWLPLF	2277.2430	1139.1251	**759.7525**	570.0667

Trypsin digest peptides numbered from the amino terminal of the polypeptide chain. * Glycopeptide. The LC-MS observed ions were indicated in bold.

Table S2. The specific ricin tryptic peptides under direct digestion.

T#&chain	Amino acid sequence	(M + H)+	(M + 2H)2+	(M + 3H)3+	(M + 4H)4+
T2A-glyc*	QYPIINFTTAGATVQSYTNFIR	3675.6946	1838.3509	**1225.9031**	919.6791
T7A	VGLPINQR	896.5312	**448.7692**	299.5152	-
T8A	FILVELSNHAELSVTLALDVTN AYVVGYR	3206.7095	1603.8584	1069.5747	**802.4328**
T9A	AGNSAYFFHPDNQEDAEAITH LFTDVQNR	3307.5038	1654.2555	1103.1728	**827.6314**
T10A	YTFAFGGNYDR	1310.5800	**655.7936**	437.5315	-
T11A	LEQLAGNLR	1013.5738	**507.2905**	338.5294	-
T12A	ENIELGNGPLEEAISALYYYSTG GTQLPTLAR	3440.7219	1720.8646	**1147.5788**	860.9359
T13A	SFIICIQMISEAAR	1581.8127	**791.4100**	527.9424	-
T23A	FSVYDVSILIPIIALMVYR	2212.2450	1106.6261	**738.0865**	553.8169
T24A-ss-T1B[a]	CAPPPSSQF-ss-ADVCMDPEPIVR	2275.0196	1138.0138	**759.0118**	569.5108

T3B-ss-T5B[a]	NGLCVDVR-ss-FHNGNAIQLWPCK	2400.1699	1200.5886	**800.7281**	600.7979
T6B	SNTDANQLWTLK	1390.6961	**695.8517**	464.2369	-
T10B[a]	CLTTYGYSPGVYVMIYDCNTAATDATR	2947.2947	1474.1510	**983.1031**	737.5791
T11B-gly*	WQIWDNGTIINPR	2991.2987	1496.1530	**997.7710**	748.5801
T12B	SSLVLAATSGNSGTTLTVQTNIYAVSQGWLPTNNTQPFVTTIVGLYGLCLQANSGQVWIEDCSSEK	6933.4237	3467.2155	2311.8127	1734.1119
T14B-ss-T16B[a]	DNCLTSDSNIR-ss-ILSCGPASSGQR	2410.1085	1205.5579	**804.0410**	603.2826
T18B	NDGTILNLYSGLVLDVR	1862.0018	931.5045	**621.3388**	466.2564
T20B	QIILYPLHGDPNQIWLPLF	2277.2430	1139.1251	**759.7525**	570.0667

Trypsin digest peptides numbered from the amino terminal of the polypeptide chain. a: Disulfide bund peptide. * Glycopeptide. The LC-MS observed ions were indicated in bold.

Table S3. Theoretical mass to charge values of ricin digested peptides from Glu-C after trypsin.

T#&chain	Amino acid sequence	$(M + H)^{+}$	$(M + 2H)^{2+}$	$(M + 3H)^{3+}$	$(M + 4H)^{4+}$
TG1A	IFPK	504.3180	252.6626	-	-
TG2A-glyc*	QYPIINFTTAGATVQSYTNFIR	3675.6946	1838.3509	**1225.9031**	919.6791
TG3A	AVR	345.2245	-	-	-
TG4A	GR	232.1404	-	-	-
TG5A	LTTGAD	577.2828	289.1450	-	-
TG6A	VR	274.1873	-	-	-
TG7A	HE	285.1193	-	-	-
TG8A	IPVLPNR	808.5039	404.7556	270.1728	-
TG9A	VGLPINQR	896.5312	**448.7692**	299.5152	-
TG10A	FILVE	620.3654	310.6863	-	-
TG11A	LSNHAE	670.3155	355.6614	-	-
TG12A	LSVTLALD	831.4822	416.2447	277.8322	-
TG13A	VTNAYVVGYR	1141.6000	**571.3036**	381.2048	-
TG14A	AGNSAYFFHPD	1225.5272	613.2672	409.1805	307.1372
TG15A	NQE	390.1619	-	-	-
TG16A	D	134.0448	-	-	-
TG17A	AE	219.0975	-	-	-
TG18A	AITHLFTD	917.4727	459.2340	306.4957	-
TG19A	VQNR	516.2889	258.6481	-	-
TG20A	YTFAFGGNYD	1154.4789	**577.7431**	385.4978	-
TG21A	R	175.1189	-	-	-
TG22A	LE	261.1445	-	-	-
TG23A	QLAGNLR	771.4471	359.2272	-	-
TG24A	E	148.0604	-	-	-
TG25A	NIE	375.1874	-	-	-
TG26A	LGNGPLE	**699.3671**	350.1872	233.7939	-
TG27A	E	148.0604	-	-	-
TG28A	AISALYYYSTGGTQLPTLAR	2146.1179	1073.5626	**716.0441**	
TG29A	SFIICIQMISE	1283.6374	642.3223	**428.5506**	-
TG30A	AAR	317.1932	-	-	-
TG31A	FQYIE	699.3348	350.1710	-	-
TG32A	GE	205.0819	-	-	-
TG33A	MR	306.1594	-	-	-
TG34A	TR	276.1666	-	-	-
TG35A	IR	288.2030	-	-	-
TG36A	YNR	452.2252	-	-	-
TG37A	R	175.1189	-	-	-
TG38A	SAPD	389.1667	-	-	-
TG39A	PSVITLE	758.4294	379.7183	-	-

TG40A	NSWGR	619.2947	310.1510	-	-
TG41A	LSTAIQE	761.4039	381.2056	-	-
TG42A	SNQGAFASPIQLQR	1516.7866	758.8969	506.2670	-
TG43A	R	175.1189	-	-	-
TG44A	NGSK	405.2092	-	-	-
TG45A	FSVYD	630.2769	315.6420	-	-
TG46A	VSILIPIIALMVYR	1600.9858	800.9965	534.3334	
TG47A-ss-TG2B[a]	CAPPPSSQF-ss-VCMD	1397.5535	**699.2804**	466.5226	-
TG1B	AD	205.0819	-	-	-
TG3B	PE	245.1132	-	-	-
TG4B	PIVR	484.3242	-	-	-
TG5B	IVGR	444.2929	-	-	-
TG6B-ss-TG9B[a]	NGLCVD-ss-FHNGNAIQLWPCK	2145.0003	1073.0038	**715.7154**	537.0555
TG7B	VR	274.1873	-	-	-
TG8B	DGR	347.1673	-	-	-
TG10B	SNTD	436.1674	-	-	-
TG11B	ANQLWTLK	973.5465	**487.2769**	325.1870	
TG12B	R	175.1189	-	-	-
TG13B	DNTIR	618.3205	309.6639	-	-
TG14B	SNGK	405.2092	-	-	-
TG15B-ss-TG16B[a]	CLTTYGYSPGVYVMIYD-ss-CNTAATD	2637.1194	1319.0633	879.7113	660.0353
TG17B	ATR	347.2037	-	-	-
TG18B	WQIWD	747.3460	347.1766	-	-
TG19B	NGTIINPR	884.4948	442.7510	295.5031	-
TG20B	SSLVLAATSGNSGTTLTVQTNIYAVSQGWLPTNNTQPFVTTIVGLYGLCLQANSGQVWIED	6284.1859	3142.5966	2095.4002	1571.8024
TG21B	CSSE	425.1337	-	-	-
TG22B	K	147.1128	-	-	-
TG23B	AE	219.0975	-	-	-
TG24B	QQWALYAD	994.4628	497.7350	332.1591	-
TG25B	GSIRPQQNR	1055.5704	528.2888	352.5283	-
TG26B	D	134.0448	-	-	-
TG27B-ss-TG30B[a]	NCLTSD-ss-ILSCGPASSGQR	1824.7458	912.9094	608.9639	**456.9912**
TG28B	SNIR	489.2780	-	-	-
TG29B	ETVVK	575.3399	288.1736	-	-
TG31B	WMFK	611.3010	306.1541	-	-
TG32B	ND	248.0877	-	-	-
TG33B	GTILNLYSGLVLD	1377.7624	689.3848	**459.9256**	
TG34B	VR	274.1873	-	-	-
TG35B	ASD	292.1139	-	-	-
TG36B	PSLK	444.2816	-	-	-
TG37B	QIILYPLHGD	1168.6360	**584.8217**	390.2169	-
TG38B	PNQIWLPLF	1127.6248	**564.3160**	376.5464	-

Trypsin/Glu-C tandem digest peptides numbered from the amino terminal of the polypeptide chain. [a]: Disulfide bund peptide. * Glycopeptide. Ricin specific peptide was indicated in red color. The LC-MS observed ions of ricin specific peptides were indicated in bold.

Table S4. The ricin specific peptides under pepsin digestion at pH 1~2.

P#&chain	Amino acid sequence	$(M + H)^{+}$	$(M + 2H)^{2+}$	$(M + 3H)^{3+}$	$(M + 4H)^{4+}$
P3A	TTAGATVQSYTNF	1360.6379	680.8226	**454.2175**	340.9154
P4A	IRAVRGRL	940.6163	470.8118	**314.2103**	-
P7A	PINQRF	**774.4257**	387.7165	258.8134	-
P13A	DVTNAYVVGYRAGNSAYF	1966.9293	983.9683	**656.3146**	492.4883
P17A	TDVQNRYTF	1143.5429	**572.2751**	381.8525	-

P19A	GGNYDRL	794.3791	397.6932	265.4646	-
P24A	EEAISAL	**732.3774**	366.6923	244.7973	-
P25A	YYYSTGGTQL	**1152.5208**	576.7640	384.8451	-
P28A	IICIQMISEAARF	1494.7807	747.8940	498.9317	374.4511
P34A	SVYDVSIL	895.4771	448.2422	299.1639	-
P36A-ss-P1B[a]	MVYRCAPPPSSQF-ss-NADVCMDPEPIVRIVGRNGL	3533.7164	1767.3622	1178.5774	884.1850
P2B-ss-P4B[a]	CVDVRDGRF-ss-WPCKSNTDANQL	2440.1136	1220.5608	814.0431	**610.7843**
P3B	HNGNAIQL	866.4479	**433.7276**	289.4875	-
P6B	KRDNTIRSNGKCL	1504.8012	752.9043	**502.2719**	376.9562
P7B	TTYGYSPGVYVMIYDCNTAAT DATRWQIWD NGTIINPRSSL	4614.1704	2307.5888	1538.7283	1154.2985
P9B	AATSGNSGTTL	979.4691	**490.2382**	**327.1612**	-
P15B	QANSGQVWIEDCSSEKAEQQ WAL	2607.1780	1304.0926	**869.7308**	652.5504
P23B	DVRASDPSL	959.4792	**480.2432**	320.4979	-
P24B	KQIIL	614.4235	307.7154	**205.4794**	-
P26B	HGDPNQIWL	**1079.5268**	**540.2671**	360.5138	-

Pepsin digest peptides numbered from the amino terminal of the polypeptide chain. a: Disulfide bound peptide. The LC-MS observed ions were indicated in bold.

Table S5. The ricin specific peptides under pepsin digestion at pH 2~4.

P#&chain	Amino acid sequence	$(M + H)^{+}$	$(M + 2H)^{2+}$	$(M + 3H)^{3+}$	$(M + 4H)^{4+}$
P2B-ss-P4B[a]	DVCMDPE-ss-CVDVRDGRF	1871.7725	936.3902	**624.5961**	468.6991
P9B	PCKSNTDA	835.3614	**418.1843**	279.1253	-
P14B	KRDNTIRSNGKCL	1504.8012	752.9043	**502.2719**	-
P25B	DNGTIINPRSSL	1286.6699	643.8386	**429.5615**	322.4234
P29B	TSGNSGTTL	837.3948	419.2011	**279.8031**	-
P60B	TSDSNIRE	921.4272	**461.2172**	307.8139	-
P77B	HGDPNQ	667.2794	**334.1433**	223.0980	-

Pepsin digest peptides numbered from the amino terminal of the polypeptide chain. a: Disulfide bound peptide. The LC-MS observed ions were indicated in bold.

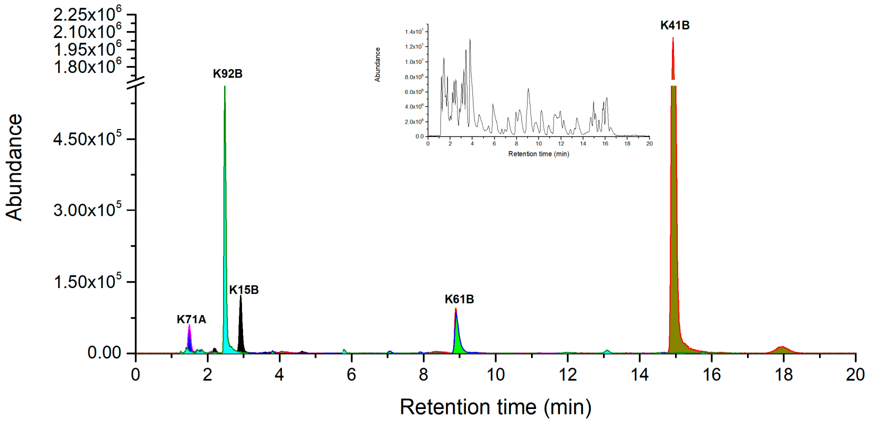

Figure S1. LC-high resolution accurate mass analysis of the proteinase K digest of 0.1 mg/mL of purified ricin. Base peak chromatogram overlaid on extracted ion chromatograms of ricin maker peptides. Total ion chromatography of ricin at top right corner window.

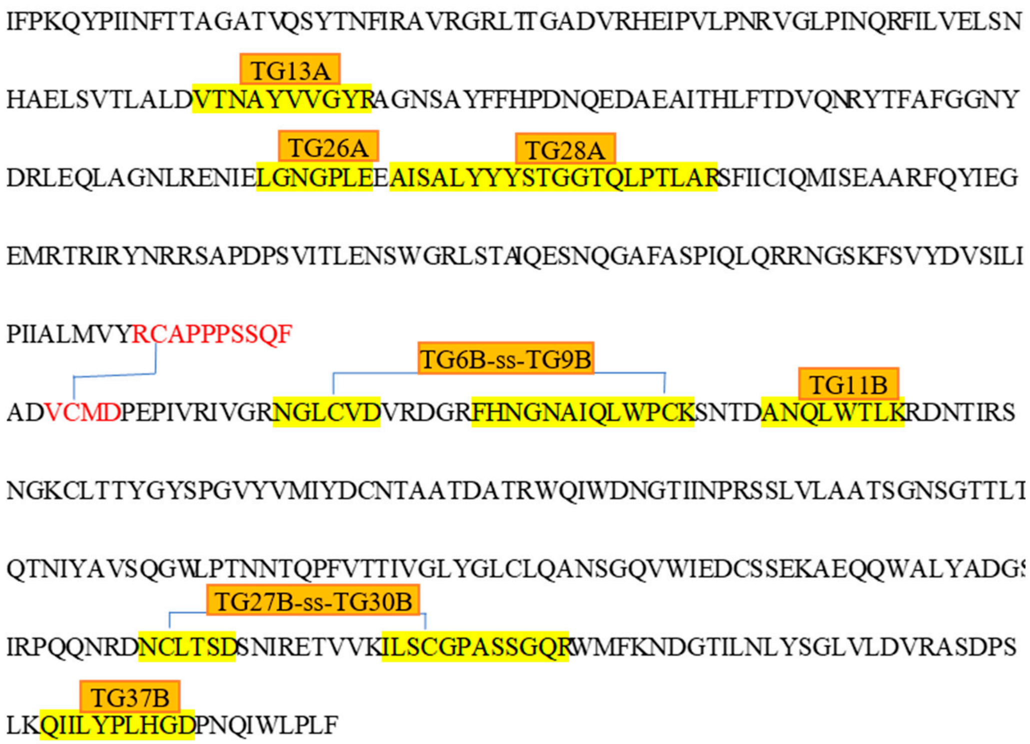

Figure S2. Peptide markers from trypsin/Glu-C tandem digestion.

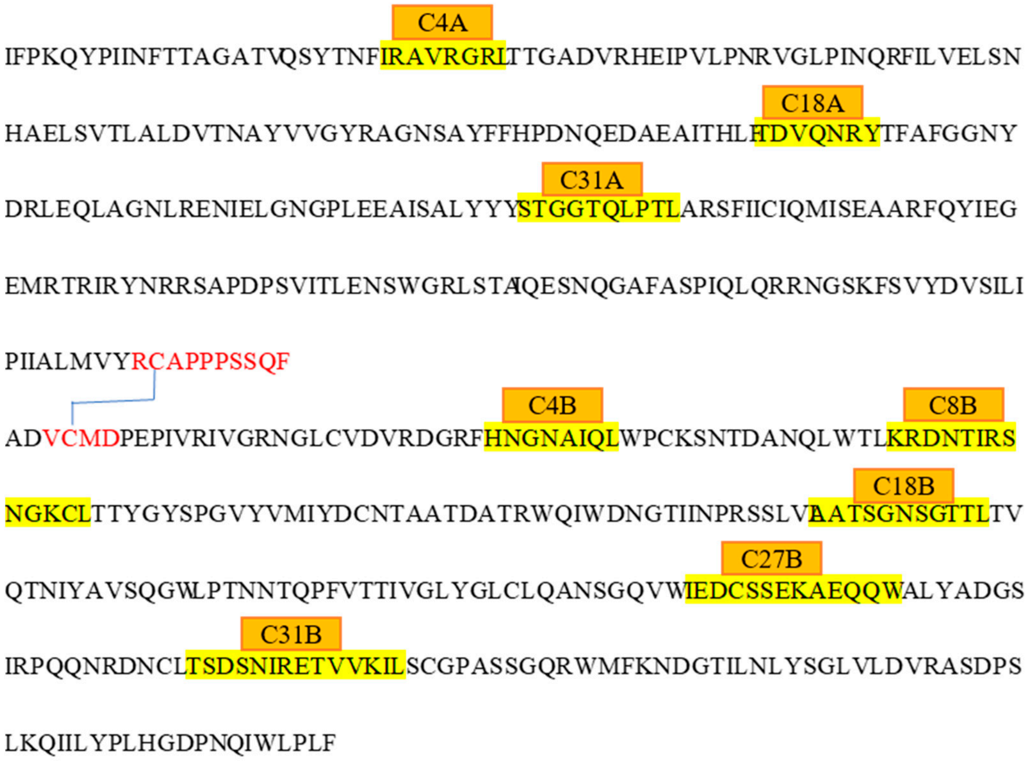

Figure S3. Peptide markers from chymotrypsin digestion.

P7A

IFPKQYPIINFTTAGATVQSYTNFIRAVRGRLTTGADVRHEIPVLPNRVGLPINQRFILVELSN

P13A

HAELSVTLALDVTNAYVVGYRAGNSAYFFHPDNQEDAEAITHLFTDVQNRYTFAFGGNY

P24A

DRLEQLAGNLRENIELGNGPLEEAISALYYYSTGGTQLPTLARSFIICIQMISEAARFQYIEG

EMRTRIRYNRRSAPDPSVITLENSWGRLSTAIQESNQGAFASPIQLQRRNGSKFSVYDVSILI

PIIALMVYRCAPPPSSQF

P6B

ADVCMDPEPIVRIVGRNGLCVDVRDGRFHNGNAIQLWPCKSNTDANQLWTLKRDNTIRS

NGKCLTTYGYSPGVYVMIYDCNTAATDATRWQIWDNGTIINPRSSLVLAATSGNSGTTLTV

QTNIYAVSQGWLPTNNTQPFVTTIVGLYGLCLQANSGQVWIEDCSSEKAEQQWALYADGS

P23B

IRPQQNRDNCLTSDSNIRETVVKILSCGPASSGQRWMFKNDGTILNLYSGLVLDVRASDPS

LKQIILYPLHGDPNQIWLPLF

Figure S4. Peptide markers from pepsin digestion at pH 1~2.

IFPKQYPIINFTTAGATVQSYTNFIRAVRGRLTTGADVRHEIPVLPNRVGLPINQRFILVELSN

HAELSVTLALDVTNAYVVGYRAGNSAYFFHPDNQEDAEAITHLFTDVQNRYTFAFGGNY

K71A

DRLEQLAGNLRENIELGNGPLEEAISALYYYSTGGTQLPTLARSFIICIQMISEAARFQYIEG

EMRTRIRYNRRSAPDPSVITLENSWGRLSTAIQESNQGAFASPIQLQRRNGSKFSVYDVSILI

PIIALMVYRCAPPPSSQF

K15B

ADVCMDPEPIVRIVGRNGLCVDVRDGRFHNGNAIQLWPCKSNTDANQLWTLKRDNTIRS

K41B

NGKCLTTYGYSPGVYVMIYDCNTAATDATRWQIWDNGTIINPRSSLVLAATSGNSGTTLTV

K61B

QTNIYAVSQGWLPTNNTQPFVTTIVGLYGLCLQANSGQVWIEDCSSEKAEQQWALYADGS

IRPQQNRDNCLTSDSNIRETVVKILSCGPASSGQRWMFKNDGTILNLYSGLVLDVRASDPS

K92B

LKQIILYPLHGDPNQIWLPLF

Figure S5. Peptide markers from proteinase K digestion.

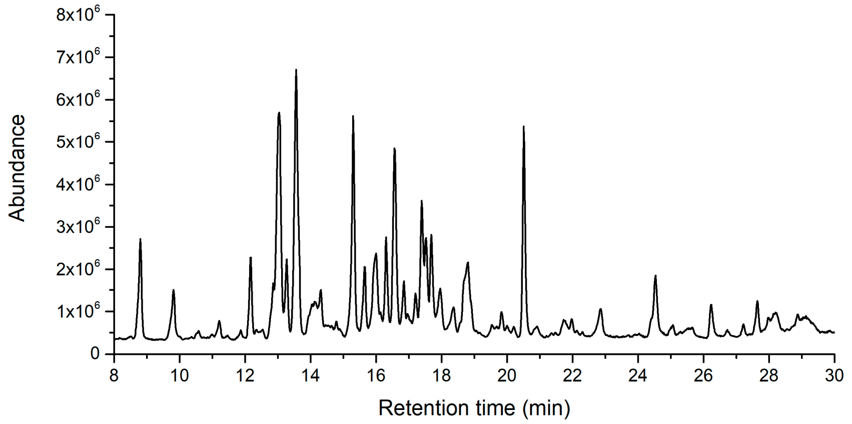

Figure S6. Total ion chromatography of LC-high resolution accurate mass analysis of the trypsin combined with Glu-C digest of 0.1 mg/mL of purified ricin.

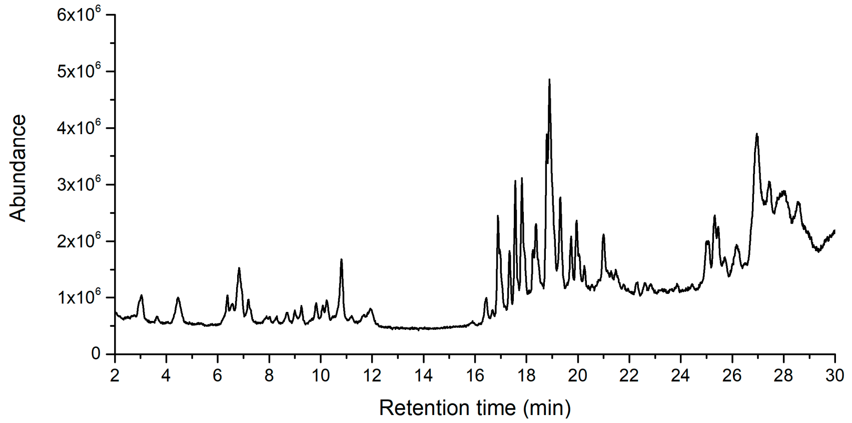

Figure S7. Total ion chromatography of LC-high resolution accurate mass analysis of the chymotrypsin digest of 0.1 mg/mL of purified ricin under denaturation and reduction.

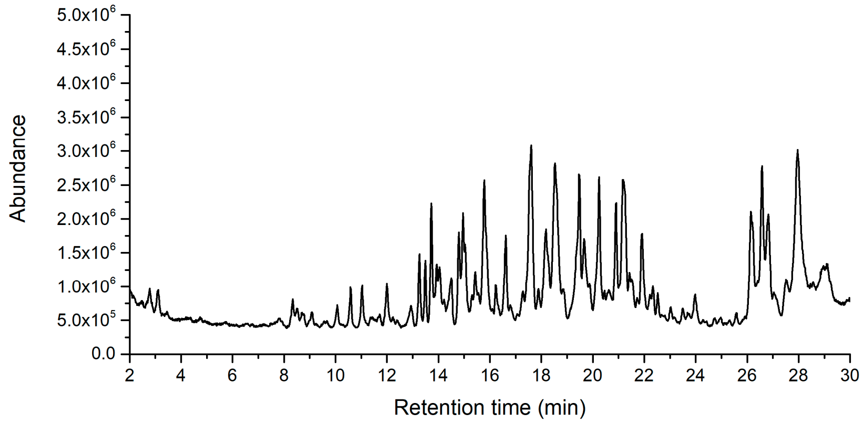

Figure S8. Total ion chromatography of LC-high resolution accurate mass analysis of the direct chymotrypsin digestion of 0.1 mg/mL of purified ricin.

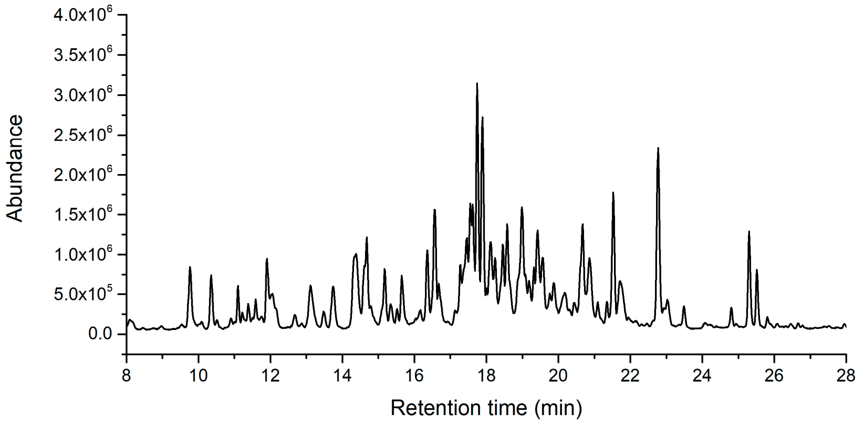

Figure S9. Total ion chromatography of LC-high resolution accurate mass analysis of the pepsin digestion of 0.1 mg/mL of purified ricin (pH 1~2).
